# Supplementary material for: Causal Relationship between Aging and Anorexia Nervosa: A White-Matter-Microstructure-Mediated Mendelian Randomization Analysis
Source: Biomedicines. 2024 Aug 16;12(8):1874. doi: 10.3390/biomedicines12081874 (PMC11351342; doi:10.3390/biomedicines12081874)
Supplement: Supplementary file 1 [file biomedicines-12-01874-s001.zip › Supplementary Figure/Figure S9/Leaveoneout plot of MR analysis for ubm-b-1969 to AN.pdf]

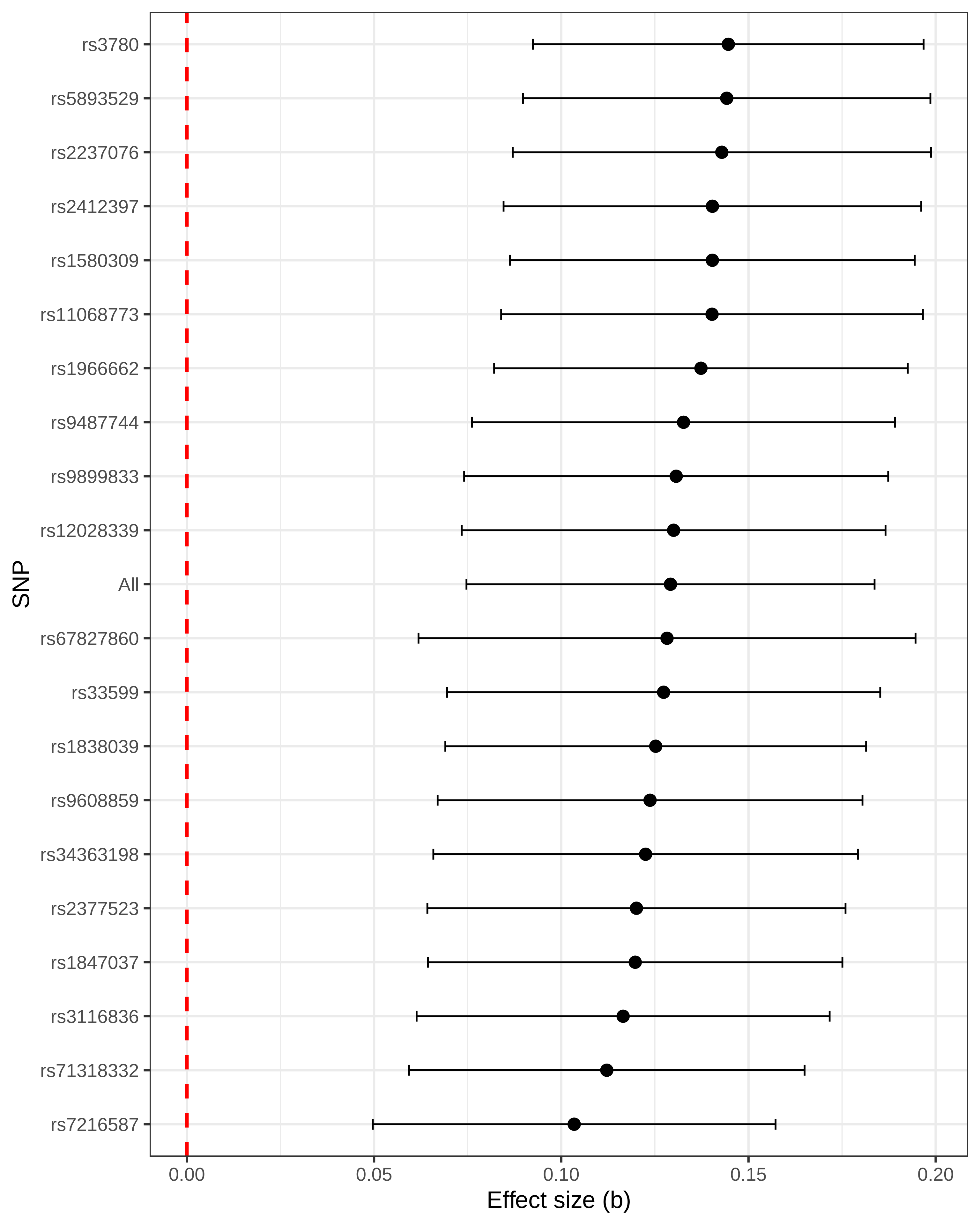

rs3780
rs5893529
rs2237076
rs2412397
rs1580309
rs11068773
rs1966662
rs9487744
rs9899833
rs12028339
All
rs67827860
rs33599
rs1838039
rs9608859
rs34363198
rs2377523
rs1847037
rs3116836
rs71318332
rs7216587
SNP
0.00
0.05
0.10
0.15
0.20
Effect size (b)
